# Comprehensive review of mitochondrial nephropathy—a renal phenotype in mitochondrial disease: causative genes, clinical and pathological features, diagnosis, prognosis, and treatment

**DOI:** 10.1007/s10157-024-02554-y

**Published:** 2024-12-03

**Authors:** Toshiyuki Imasawa, Kei Murayama, Daishi Hirano, Kandai Nozu

**Affiliations:** 1 Department of Nephrology, National Hospital Organization Chibahigashi National Hospital, 673 Nitona-cho, Chuoh-ku, Chiba, 206-8712 Japan; 2 Diagnostics and Therapeutic of Intractable Diseases, Intractable Disease Research Center, Graduate School of Medicine, Juntendo University, 2-1-1, Hongo, Bunkyo-ku, Tokyo, 113-8421 Japan; 3 Department of Pediatrics, The Jikei University School of Medicine, 3-25-8 Nishi-Shinbashi, Minato-ku, Tokyo, 105-0003 Japan; 4 Department of Pediatrics, Kobe University Graduate School of Medicine, Kobe, Hyogo 650-0017 Japan

**Keywords:** Mitochondrial nephropathy, Mitochondria, Focal segmental glomerulosclerosis, Tubulointerstitial nephropathy, Granular swollen epithelial cell

## Abstract

Mitochondrial nephropathy is a genetic renal disease characterized by oxidative phosphorylation abnormalities in the mitochondrial respiratory chain in kidney cells, caused by pathogenic gene variants located on mitochondrial or nuclear DNA. Recent advancements in genetic diagnostic techniques and their widespread adoption have led to the identification of various genes associated with mitochondrial nephropathy. This review investigates the causative genes and clinicopathological features of mitochondrial nephropathy, including the various phenotypes and associated complications, and suggests potential pathogenic mechanisms. Furthermore, the diagnostic methods of the disease are explained with particular emphasis on characteristic pathological findings and genetic analysis. We also analyze the available long-term observational prognostic data. Although there is currently no evidence-based treatment for mitochondrial nephropathy, an overview of the existing treatment options is discussed, including future expectations. The choice of renal replacement therapy in cases with progression to end-stage renal disease has also been discussed. Overall, this review highlights the importance of raising awareness about mitochondrial nephropathy and establishing appropriate diagnostic systems to facilitate rapid and effective treatment.

## Introduction

The mitochondrial respiratory chain (MRC) is the fundamental structure facilitating intracellular oxidative phosphorylation (OXPHOS) and is essential for cellular energy metabolism. The function of MRC is governed by genes encoded both in mitochondrial DNA (mtDNA) and nuclear DNA (nDNA) [1, 2]. Pathogenic variations in these genes cause abnormalities in MRC-mediated OXPHOS, promoting mitochondrial disease development.

The role of mtDNA abnormalities in human pathological conditions was first established in 1988 [3, 4]. A plethora of pathogenic mtDNA variants have since been identified in mitochondrial diseases, which are comprehensively cataloged in the MITOMAP human mitochondrial genome database (http://www.mitomap.org). A number of mitochondrial diseases have been also associated with pathogenic variants of nDNA, which carries more causative genes than those on mtDNA [1, 2].

Mitochondrial diseases induce a range of dysfunctions in multiple organs, including the kidneys [5]. To facilitate the accurate diagnosis of mitochondrial nephropathy—a renal phenotype in mitochondrial disease—this review investigated the causative genes, clinical and pathological features, etiology, and potential diagnostic strategies. No double-blind placebo-controlled trials have been performed in mitochondrial nephropathy; however, some candidate drugs have been identified. Therefore, we also described the prognosis and treatment of mitochondrial nephropathy. Through our review of the current paradigm in mitochondrial nephropathy research, we also highlight current issues that hinder advances in the field.

## Mitochondrial nephropathy

Mitochondrial nephropathy is a genetic renal disease caused by abnormalities in MRC-mediated OXPHOS in kidney cells due to pathogenic gene variants located on mtDNA or nDNA [6]. Although no definitive classification exists, mitochondrial nephropathy has been described as a “renal involvement in mitochondrial cytopathy” [5, 7], “renal manifestations of mitochondrial disorders” [8], and renal phenotype in mitochondrial disease [9]. However, despite their phenotypic diversity, mitochondrial nephropathies share a common pathogenesis, i.e., genetically impaired mitochondrial function in kidney cells, as well as common clinical features and pathological findings. This indicates the possibility for common diagnostic and therapeutic approaches. Therefore, classifying mitochondrial nephropathy as an independent disease entity, such as mitochondrial cardiomyopathy and hepatopathy [10, 11], may improve the diagnosis and prognosis of patients.

Owing to the potential occurrence of nephropathy secondary to non-renal organ damage caused by mitochondrial disease [12–14], it is necessary to differentiate between mitochondrial nephropathy and nephropathy secondary to mitochondrial disease in selecting appropriate treatment.

## Causal genes

The association between pathogenic gene variants on mtDNA and kidney diseases was first documented in the 1990s. Focal segmental glomerulosclerosis (FSGS), Fanconi syndrome, and tubulointerstitial nephropathy have been associated with pathogenic gene variants encoding mitochondrial transfer RNAs (mt-tRNAs) [15–19]. Pathogenic variants of *MT-TI* and *MT-TF* can cause hypokalemia and hypomagnesemia, which are similar to Gitelman-like syndrome [20]. Around the same time, single deletions in mtDNA were shown to cause Bartter-like syndrome, tubular acidosis, and FSGS [21–23]. After 2006, nDNA-encoded homozygous or compound heterozygous pathogenic gene variants related to coenzyme Q10 (CoQ10) synthesis were reported to cause steroid resistant nephrotic syndrome (SRNS) and FSGS [24–28]. After 2018, mitochondrial nephropathy caused by pathogenic variants on mtDNA coding for subunits of respiratory chain complex V (ATP synthase), complex IV, or complex I [29–31] have been identified. Homozygous or compound heterozygous pathogenic variants of the required for meiotic nuclear division protein 1 (*RMND1*) gene—located on nDNA and coding for a mitochondrial inner membrane protein—frequently cause renal diseases, such as tubulopathy, renal tubular acidosis, interstitial nephritis, and/or end-stage renal disease [32]. Furthermore, multiple deletions in mtDNA—caused by a pathogenic variant of ribonucleotide reductase M2B (*RRM2B*) on nDNA—resulted in proximal tubulopathy [33]. A recent Japanese case report described a developmental disorder primarily caused by a pathological variant of *BCS1L* on nDNA, resulting in MRC complex III deficiency and Fanconi syndrome [34].

Causal genes for mitochondrial diseases can be categorized according to their function [28, 35–37]. Table 1 presents the causative genes associated with mitochondrial nephropathy/renal involvement in mitochondrial disease, categorized according to their function [38–64], along with the renal manifestations and pathologies. Although the nephropathies associated with the pathogenic variants described in Table 1 cannot be clearly distinguished as mitochondrial nephropathy or nephropathy secondary to non-renal organ damage caused by mitochondrial disease, it shows that causative genes may have various functions and mitochondrial nephropathy is phenotypically diverse.

**Table 1:** Causative genes for mitochondrial nephropathy/renal involvement in mitochondrial disease

Disease mechanism	Gene function	Mutated genes (^a^frequency in Japan)	Renal manifestations/pathology	Reference(s)
Oxidative phosphorylation deficiency	Complex I subunits and assembly factors	*MT-ND5* (*1.3%*), *NDUFAF2*, *ACAD9*	Tubular acidosis, TIN, glomerulocystic disease, mitochondrial hyperplasia in tubules	[31, 38, 39]
	Complex III subunits and assembly factors	*BCS1L*, *UQCC2*	Fanconi syndrome, tubular dysfunction	[34, 40–43]
	Complex IV subunits and assembly factors	*MT-CO1*, *SURF1*, *COX10*	Tubulopathy, TIN	[30, 44, 45]
	Complex V subunits and assembly factors	*MT-ATP6*, *TMEM70*	FSGS, TIN, tubulopathy	[29, 46, 47]
Disorders of mitochondrial DNA maintenance	Nucleotide pool maintenance	*RRM2B* (*2.5%*), *DGUOK*, *SUCLA2*, *TK2*	Fanconi syndrome, tubulopathy	[33, 48–50]
	Replication, maintenance, and transcription of mtDNA	*MPV17*	Tubulopahty	[51]
Mitochondrial translation defects	Mitochondrial tRNAs	*MT-TF*, *MT-TI*, *MT-TL1* (79.1%), *MT-TN*, *MT-TW* (1.3%), *MT-TY*, *MT-TK*	TIN, tubulopathy, Fanconi syndrome, FSGS, nephrotic syndrome	[15–20, 52]
	Mitochondrial aminoacyl-tRNA synthetases	*SARS2*, *YARS2*, *LARS2*	Tubulopathy, kidney failure	[53–56]
	Mitoribosome subunits and assembly	*RMND1*, *MRPS22*, *MRPL44*	Tubulopathy, TIN, tubular acidosis, kidney failure	[32, 57, 58]
	Protein synthesis	*TSFM*	Tubulopathy	[59]
Mitochondrial quality control defects	Mitochondrial membrane phospholipid and import machinery	*XPNPEP3*	Kidney failure	[60]
	Mitochondrial protein quality control	*CLPB*	Nephrocalcinosis	[61]
	Toxicity	*ETHE1*	Crescentic glomerulonephritis	[62]
Vitamin and cofactor metabolism defects	Coenzyme Q_10_ biosynthesis	*COQ2* (2.5%), *COQ6* (1.3%), *COQ8B* (7.4%), *COQ9*, *PDSS2*	FSGS, (steroid-resistant) nephrotic syndrome, tubular dysfunction	[24–28, 63, 64]

Causative genes on mtDNA are underlined

The frequency data is based on Ref. [6]. The total percentages do not add up to 100% because of single mtDNA deletion (3.7%) and multiple mtDNA deletions in which the causative gene was unknown (1.3%)

*TIN* tubulointerstitial nephropathy, *FSGS* focal segmental glomerulosclerosis

^a^It was not possible to clearly distinguish between nephropathy caused by the pathogenic variants or nephropathy secondary to non-renal organ damage caused by mitochondrial disease

The m.3243A>G variant in *MT-TL1* is the most frequent pathogenic gene variant in mitochondrial disease [65]. In our study of a Japanese cohort, the m.3243A>G variant was also the most common in mitochondrial nephropathy (77.8%) [6]. With advancements in gene analysis techniques and increasing awareness of mitochondrial diseases, the number of diagnoses is expected to increase and new causal pathogenic variants are likely to be identified.

## Clinical characteristics

Proximal tubular cells contain some of the highest numbers of mitochondria among cells in the body [66]; in glomeruli, mitochondria are relatively abundant in visceral epithelial cells (podocytes) [67]. Renal symptoms associated with proximal tubular cell damage and podocyte injury, such as Fanconi syndrome and FSGS, respectively, have been frequently observed in mitochondrial nephropathy. Fanconi syndrome defines a group of disorders characterized by impaired reabsorption of phosphate, uric acid, glucose, amino acids, low-molecular-weight proteins, and bicarbonate caused by proximal tubule transporter dysfunction, resulting in metabolic acidosis, electrolyte imbalance, dehydration, or rickets. Reduced glomerular filtration rates or proteinuria can manifest as renal symptoms in mitochondrial nephropathy due to the development of FSGS or nephrotic syndrome, both of which are caused by mitochondrial dysfunction in podocytes [67–69]. Patients with mitochondrial nephropathy may experience electrolyte abnormalities, such as hypokalemia or hypomagnesemia, due to distal tubular cell damage, resulting in Bartter or Gitelman syndromes. Patients with fibrotic or inflammatory lesions in the renal tubule-interstitium also presented with deteriorating glomerular filtration rates [20, 70, 71].

According to our previous nation-wide survey in Japan, patients with mitochondrial nephropathy caused by mtDNA variants presented with proteinuria (≥0.15 g/g Cre), reduced estimated glomerular filtration rate (eGFR; <60 mL/min/1.73 m^2^), or Fanconi syndrome at a median age of 20.0 years [interquartile range (IQR): 14.0–32.0 years] [6]. Drovandi et al. [72] reported that nephropathy caused by pathogenic gene variants related to CoQ10 synthesis mostly occurred in children, with median ages of 1.0, 1.2, and 9.8 years at renal disease onset in patients with pathogenic variants of *COQ2*, *COQ6*, and *COQ8B*, respectively. Notably, the onset of renal disease caused by pathogenic variants of *COQ8B* can occur in adolescence [73] or after the age of 30 [74]. In our survey, proteinuria and the decrease in eGFR were observed in 93.6% and 72.8% of mitochondrial nephropathy cases, respectively [6]. A study analyzing 74 cases of maternally inherited diabetes and deafness with the m.3243A>G variant reported renal dysfunction in 46.3% cases [75]. Another study analyzing 75 adult patients with maternally inherited diabetes and deafness (MIDD); mitochondrial encephalopathy, lactic acidosis, and stroke-like episodes (MELAS); and myoclonic epilepsy with ragged red fibers (MERRF), all with the m.3243A>G variant, reported no increase in serum creatinine levels; however, albuminuria and increased urinary retinol-binding protein levels (an indicator of tubular damage) were observed in 30.7% and 38.7% cases, respectively [76].

**Fig. 1:**
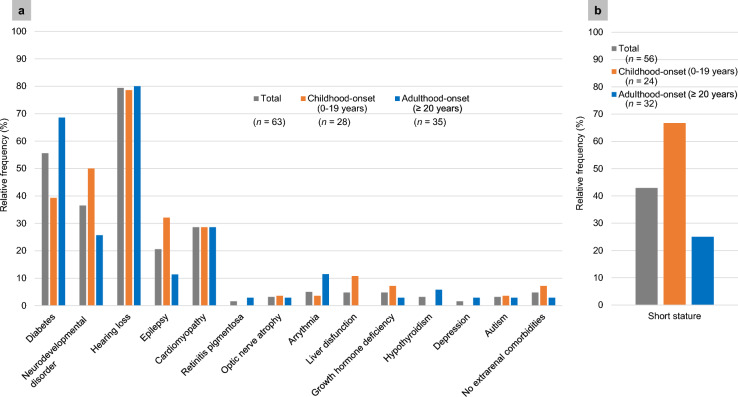
Frequency of comorbidities and short stature in cases of mitochondrial nephropathy with m.3243A>G variant. **a** Frequency of comorbidities. Frequencies are shown for childhood-onset cases (*n* = 28) and adulthood-onset cases (*n* = 35), as well as the overall frequency. Time of onset of renal manifestations was defined as the time at first detection of at least one of following renal manifestations: proteinuria (≥0.15 g/gCre), reduced eGFR (<60 mL/min/1.73 m^2^), or Fanconi syndrome. This figure is based on data from Ref. [6]. **b** Frequency of short stature. These results were obtained by analyzing data from 56 (24 childhood-onset and 32 adulthood-onset cases) of the 63 cases for which height data were available in Ref. [6]

In our previous survey, >10% of patients with m.3243A>G variants presented with hearing loss, diabetes, neurodevelopmental disorders, cardiomyopathy, and epileptic seizures (Fig. 1a). Although less frequent, other complications included retinitis pigmentosa, optic nerve atrophy, arrhythmias, liver dysfunction, growth hormone deficiency, hypothyroidism, depression, and autism. We found that the clinical features differed between patients with disease onset in childhood and adulthood. For example, childhood-onset cases had fewer instances of diabetes but more instances of neurodevelopmental disorders compared to adult-onset cases (Fig. 1a). Notably, 5% of patients showed “renal-limited” symptoms (Fig. 1a). In contrast, mitochondrial nephropathy caused by pathogenic gene variants related to CoQ10 biosynthesis showed “renal-limited” symptoms in 34%, 10.8%, and 70.7% of cases with variants in *COQ2*, *COQ6*, and *COQ8B*, respectively [72].

We analyzed the occurrence of short stature, a common manifestation in patients with mitochondrial diseases [77], in cases of mitochondrial nephropathy with m.3243A>G variant, using data collected in a previous study [6]. To determine the standard deviation score (SDS) for height, we used the Excel-based Clinical Tools for Growth Evaluation of Children, provided by the Japanese Society of Pediatric Endocrinology (available at: http://jspe.umin.jp/medical/chart_dl.html; accessed June 11, 2023). For individuals aged 0–18 years, short stature was classified as ≤−2.0 SDS compared to reference data for healthy individuals of the same age and sex. For individuals aged ≥19 years, short stature was defined based on the School Health Statistics Survey (available at: https://www.mext.go.jp/b_menu/toukei/chousa05/hoken/kekka/k_detail/1411711_00003.html; accessed June 11, 2023): males and females with heights ≤159 cm and ≤147 cm were considered short. Re-evaluation of the data showed that 42.9% of patients (66.7% childhood-onset and 25.0% adulthood-onset cases) exhibited short stature (Fig. 1b).

Several reports have indicated the presence of renal calcification, kidney stones, urinary stones, or renal cysts in mitochondrial diseases [8, 78]; however, it is unclear whether these conditions are caused by genetic dysfunction of cells in the kidneys, urinary tract, or other organs. We previously observed hematuria in 26 of 81 cases (32.1%) of mitochondrial nephropathy and 19 of 63 cases (30.2%) having the m.3243A>G variant [79]. Therefore, this high frequency of hematuria could be due to the presence of urinary or kidney stones.

## Etiology of phenotypically diverse mitochondrial nephropathy manifestations associated with pathogenic mtDNA variants

**Fig. 2:**
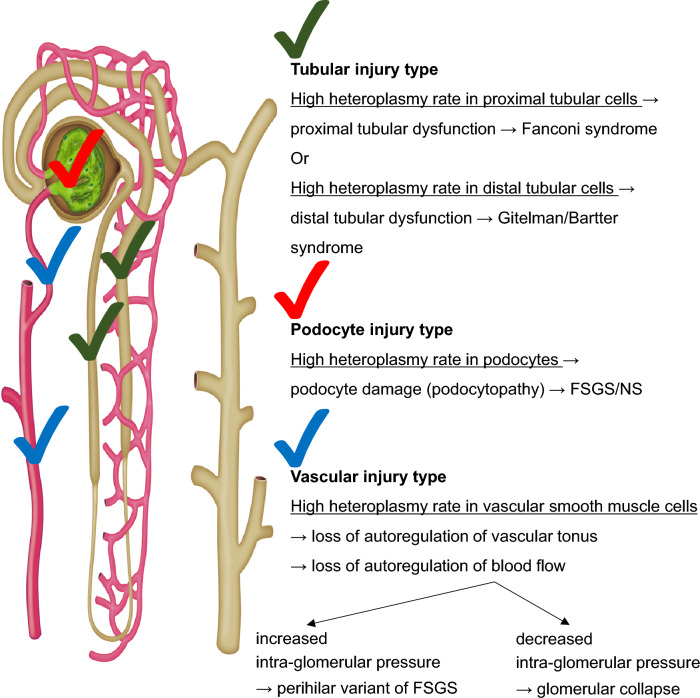
Presumed mechanisms of diverse renal manifestations in mitochondrial nephropathy caused by pathogenic mtDNA variants. FSGS, focal segmental glomerulosclerosis; NS, nephrotic syndrome

Multiple copies of mtDNA are present in each cell. Most cases of mitochondrial disease are associated with heteroplasmic mtDNA with pathogenic variants. The frequency of mtDNA with pathogenic variants (heteroplasmy rate) differ significantly between cells, tissues, and organs, resulting in the manifestation of diverse disease phenotypes [1, 2, 80]. The etiology of phenotypic diversity among mitochondrial nephropathies caused by pathogenic mtDNA variants is unclear. High heteroplasmy rates of pathogenic mtDNA variants have been observed in swelling tubular cells, which showed an accumulation of abnormal mitochondria compared with normal tubular cells [81]. Animal experiments and human case studies have shown a potential association between high heteroplasmy rates of abnormal mtDNA in glomerular epithelial cells (podocytes) and FSGS [69, 82]. These reports suggest that phenotypic diversity in mitochondrial nephropathy occurs owing to differences in the heteroplasmy rates of pathogenic mtDNA variants between kidney segments (Fig. 2). For instance, cases with high pathogenic variant rates in podocytes may present with FSGS or nephrotic syndrome, while those in proximal or distal tubular cells may present with Fanconi syndrome or Gitelman/Bartter syndrome-like symptoms, respectively, due to tubular dysfunction (Fig. 2). Furthermore, high pathogenic variant rates in vascular smooth muscle cells may cause the loss of vascular tonus autoregulation, resulting in increased/decreased intraglomerular blood pressure and possibly resulting in perihilar variants of FSGS/glomerular collapse (Fig. 2). Kidney tissue damage or renal dysfunction are not caused by impairment of a single kidney cell or segment, but by various cellular and segmental abnormalities. Therefore, the mechanisms underlying the diverse phenotypic presentations of mitochondrial diseases are likely more complex than current knowledge suggest. Therefore, the relationship between the heteroplasmy rates of pathogenic mtDNA variants in various kidney segments and the manifestations of nephropathy requires further investigation.

## When should mitochondrial nephropathy be suspected?

The diagnosis of mitochondrial nephropathy can be made in two ways: (1) when renal manifestations occur in previously diagnosed cases, or (2) when renal manifestations occur in previously undiagnosed cases of mitochondrial disease.

### In previously diagnosed cases of mitochondrial disease

In cases of mitochondrial diseases, regular measurement of the GFR, proteinuria, and serum electrolyte abnormalities is essential to evaluate renal function. In routine clinical practice, eGFR values are usually calculated from serum creatinine levels; however, in cases with low muscle mass due to mitochondrial myopathy or short stature, the serum creatinine level may not rise because of low creatinine production from muscles, which can potentially lead to GFR overestimation [83]. In such cases, GFR should be estimated using serum cystatin C.

Due to proximal tubular dysfunction in mitochondrial nephropathy, patients may develop aminoaciduria, glycosuria, hypokalemia, hypophosphatemia, hypouricemia, and renal tubular acidosis. Distal tubular cell dysfunction may further cause hypomagnesemia and hypokalemia [70, 84]. A recent systematic review indicated that 289 of 362 patients (79.8%) with mitochondrial diseases had an electrolyte disorder and frequently presented with calcium, magnesium, potassium, and sodium deficiencies [84]. Therefore, electrolyte abnormalities observed during patient follow-up may indicate the development of mitochondrial nephropathy. However, these cases of electrolyte abnormalities are not only caused by proximal/distal tubular dysfunction due to mitochondrial nephropathy, but can also be induced by parathyroid dysfunction, gastrointestinal disorders, and iatrogenic causes [84].

### In undiagnosed cases of mitochondrial disease

The opportunity to suspect mitochondrial nephropathy lies in the presence of extrarenal symptoms observed in mitochondrial disease described before (Fig. 1) in addition to renal manifestations. However, certain cases are “kidney-limited” where there are no extrarenal symptoms. In either case, renal biopsy pathological findings serve as an important trigger to suspect mitochondrial nephropathy and proceed to genetic testing.

FSGS is a key renal manifestation in mitochondrial nephropathy [6, 82]. A retrospective review of seven FSGS cases with unknown causes reported the m.3243A>G variant in four cases [85]. Some cases of mitochondrial nephropathy were diagnosed after genetic testing for SRNS [70]. Therefore, in cases of FSGS or SRNS of unknown etiology, genetic renal diseases, including mitochondrial nephropathy, may represent the underlying cause. Furthermore, mitochondrial nephropathy may be latent in patients diagnosed with nephrosclerosis, diabetic nephropathy, tubulointerstitial renal disorders, and minor glomerular abnormalities [6]. Not all cases with these pathological diagnoses need to be suspected of mitochondrial nephropathy, but the coexistence of systemic symptoms associated with mitochondrial disease, as well as family history, may raise the possibility of mitochondrial nephropathy. Alternatively, characteristic pathological changes in renal cells caused by pathological variants in both nDNA and mtDNA may also indicate mitochondrial nephropathy.

## Pathological findings in renal cells suspected of mitochondrial nephropathy

### Electron microscopy findings

In mitochondrial diseases, abnormal mitochondria are commonly observed within cells of the affected organs, with either irregular shapes or the partial/complete disruption of the mitochondrial inner membrane cristae [86–88], which may be fragmented, disoriented, or concentric [86–88]. Mitochondria of a wide range of shapes and sizes (from shrunken to enlarged) may accumulate in the affected cells. Distended mitochondria filled with a dense accumulated matrix in place of the cristae may also be observed [86–88].

**Fig. 3:**
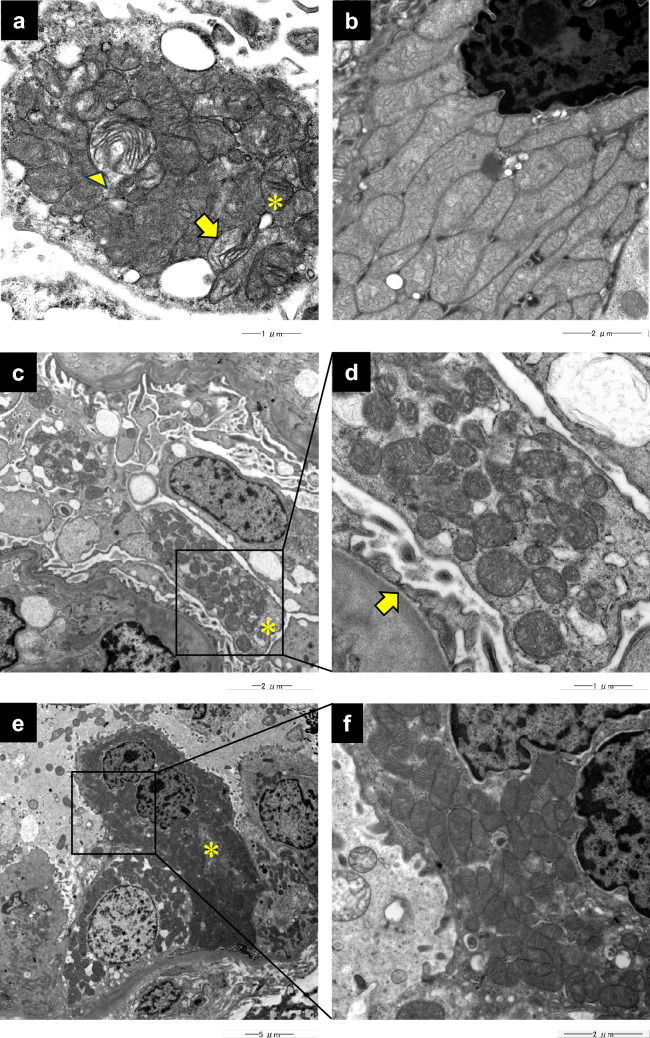
Representative electron microscopy images in mitochondrial nephropathy. **a** Abnormal mitochondria in a podocyte. Fragmented (*), concentric-like (arrow head), and partially disrupted (arrow) cristae are observed. **b** Abnormal mitochondria in a collecting duct cell. Disoriented cristae are accumulated. **c** Abnormal accumulation of mitochondria in podocytes (*). **d** Enlarged inset from **c**; accumulated mitochondria are swollen and cristae of some mitochondria are lost or torn, resulting in an abnormal mitochondrial shape. Podocyte foot process effacement is indicated by an arrow. **e** Swollen collecting duct cell (*) with abnormal accumulation of mitochondria. **f** Enlarged inset from **e**; cristae are irregularly arranged, lost, or torn

Similarly, in mitochondrial nephropathy, the accumulation of abnormal mitochondria can be observed in the podocytes and tubular cells under electron microscopy [81, 89]. Figure 3a and b show representative images of abnormal mitochondria in a podocyte and collecting duct cell. The podocytes with increased accumulation of abnormal mitochondria likely suffer from cell damage, leading to foot process effacement (Fig. 3c, d). The accumulation of abnormal mitochondria was also observed in renal tubular cells (Fig. 3e, f); however, it may be challenging to determine abnormal increases in the proximal tubules and intercalated cells of the collecting duct, as these cells already contain numerous mitochondria [81]. Furthermore, secondary morphological changes in mitochondrial shape and cristae can occur in proximal tubules, making it difficult to judge whether such abnormal mitochondria are caused by mitochondrial nephropathy [90]. In contrast, distal tubular and principal cells of the collecting ducts have fewer mitochondria, making it easier to detect abnormal accumulations [81]. Abnormal mitochondrial accumulations may also be observed in smooth muscle cells of the arterioles and interlobular arteries [16, 91] and glomerular parietal epithelial cells of the Bowman’s capsule [92].

### Light microscopy findings

**Fig. 4:**
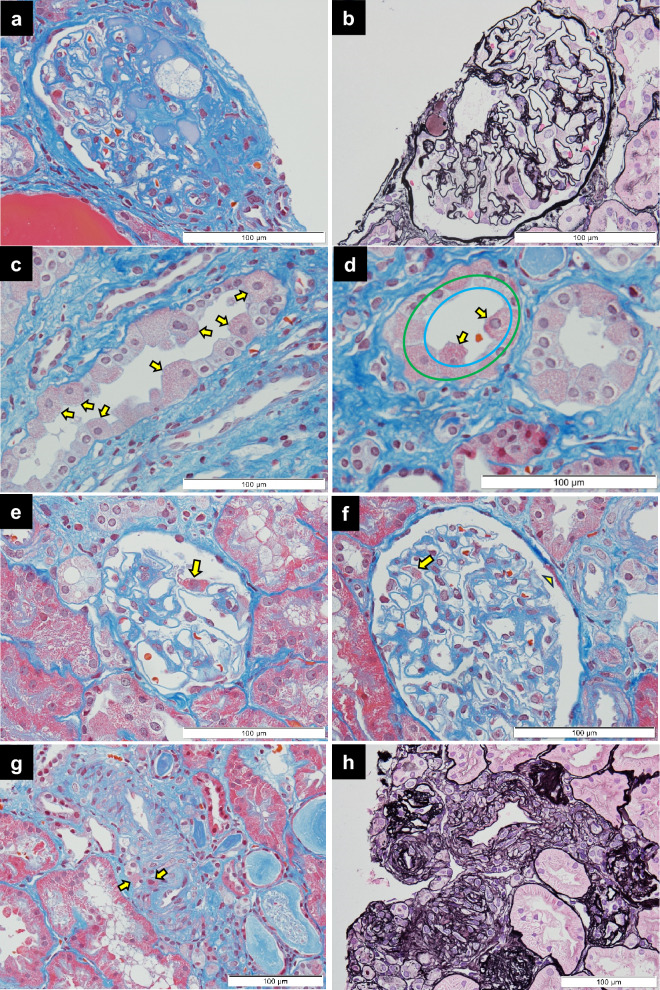
Representative images of light-microscopic findings in a female patient (in her 30 s) with mitochondrial nephropathy, having m.3243A>G variant. **a** Renal pathology indicates FSGS. **b** Hyalinotic lesion present in perihilar area of glomerulus. **c** Several GSECs (arrows) in a collecting duct. **d** GSECs (arrows) protrude into the tubular lumen from an array of other tubular cells (blue circle) and their nuclei localize further away from the tubular basement membrane compared to the nuclear arrangement of other cells (green circle). **e** Red podocyte (arrow) with abnormal accumulation of mitochondria. **f** Red podocyte (arrow) and red parietal epithelial cell present in Bowman’s capsule (arrow head). **g** Red vascular smooth muscle cells (arrows) in an interlobular artery, indicating abnormal accumulation of mitochondria. **h** Age-inappropriate disarranged and irregularly sized vascular smooth muscle cells (AiDIVs) in an interlobular artery and arteriole. **a**,**c**–**g** Masson Trichrome staining; **b**,**h** periodic acid–methenamine silver and hematoxylin–eosin (PAM-HE) staining

Mitochondrial nephropathy shows diverse renal pathologies under light microscopy [16, 81, 93–96]. FSGS is the most common presentation (Fig. 4a, b), but nephrosclerosis, diabetic nephropathy, and tubulointerstitial nephropathy can also be observed [6, 70, 81, 82, 93].

In the pathological diagnosis of mitochondrial nephropathy, it is noteworthy that mitochondria appear red when stained with acidic dyes, such as eosin and fuchsin [81]. Therefore, cells with abnormal mitochondrial accumulation appear swollen and red when stained with hematoxylin–eosin (HE), periodic acid–methenamine silver (PAM)-HE, or Masson’s trichrome, which include eosin or fuchsin (Fig. 4c). Kobayashi et al. [81] reported granular swollen epithelial cells (GSECs)—tubular cells enlarged and stained red by acidic dyes owing to accumulation of mitochondria—as a characteristic finding of mitochondrial nephropathy under light microscopy. The presence/absence of GSECs is easier to determine in cells of the collecting ducts and distal tubules because they contain fewer mitochondria [81]. Recently, cases have emerged where the presence of GSECs has led to the identification of new causative genes for mitochondrial nephropathy [30, 31]. However, it is important to note that GSECs may also occur in the kidneys of older adults, transplant kidneys, and renal diseases caused by low birth weight [97, 98]. In some cases, GSECs may be difficult to differentiate from other cells; while there is no clear criterion, GSECs typically protrude into the lumen compared to other cells (Fig. 4c, d) [81]. In addition, cell nuclei are usually located on the tubular basement membrane side; however, in GSECs, the nuclei lose their polarity and are located nearer the lumen (Fig. 4d).

Similarly, when mitochondria abnormally accumulate in podocytes, these podocytes appear red with acidic dye staining (Fig. 4e, f); some cases of mitochondrial nephropathy due to pathogenic mtDNA and nDNA variants have been diagnosed on the observation of such podocytes [74, 92]. In cases of FSGS or SRNS of unknown etiology, it is important to meticulously observe findings of swollen red podocytes by staining with acidic dyes under light microscopy, as this observation might be missed in electron microscopy due to small sample volumes. In mitochondrial nephropathy, such red-colored cells might also be observed in parietal epithelial cells of Bowman’s capsule (Fig. 4f) or vascular smooth muscle cells of the interlobular arteries or arterioles (Fig. 4g) [91, 92]. The presence of age-inappropriate disarranged and irregularly sized vascular smooth muscle cells (AiDIVs) in intralobular arteries or arterioles (Fig. 4h), without risk factors for arteriosclerosis (hypertension or dyslipidemia), commonly associated with “aged kidneys,” may also suggest mitochondrial nephropathy [91, 92].

## Diagnosis of mitochondrial nephropathy

**Fig. 5:**
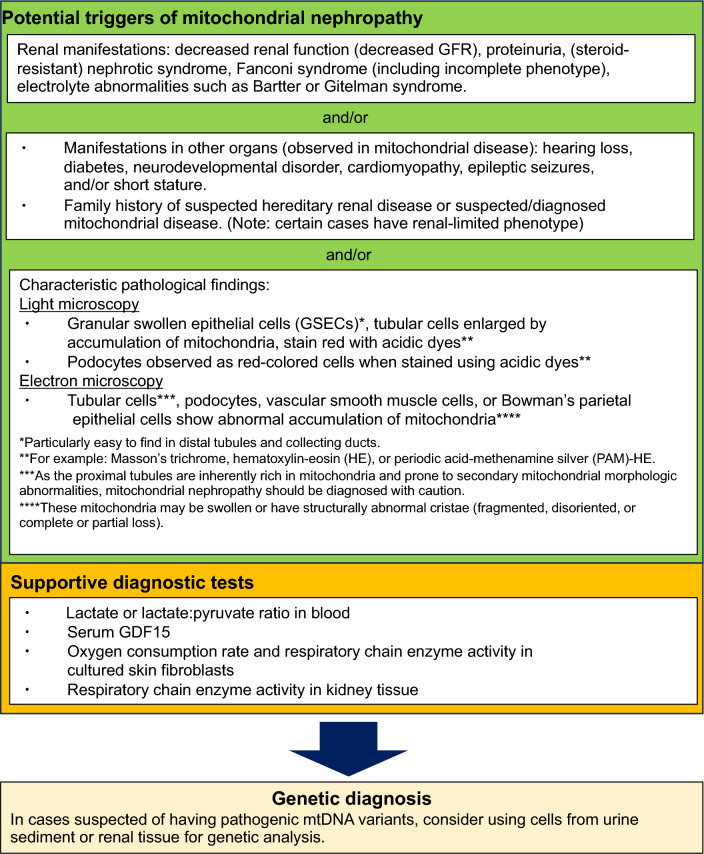
Flowchart for the diagnosis of mitochondrial nephropathy. GFR, glomerular filtration rate; GDF15, growth differentiation factor 15

In our previous survey, the median time from onset of renal manifestations to diagnosis of mitochondrial nephropathy was 6 years [6]. Given the progressive nature of renal dysfunction in mitochondrial nephropathy, it is crucial that the delay between onset and diagnosis is resolved to improve the timing and efficacy of treatments. The strategy for diagnosis of mitochondrial nephropathy is outlined in Fig. 5.

Increased levels of blood lactate or a lactate:pyruvate ratio >20 are considered indicative of mitochondrial disease [13, 36, 99]; however, we found that lactate levels were not increased in 56.2% of mitochondrial nephropathy cases and 81.5% of adult cases with the m.3243A>G variant [6]. Serum concentrations of growth differentiation factor 15 (GDF15) may also be useful for the diagnosis of mitochondrial nephropathy [100, 101].

Measuring respiratory chain enzyme activity and oxygen consumption rate using cultured fibroblasts obtained from skin biopsy is useful for diagnosis of mitochondrial diseases [102]. In addition, measuring respiratory chain enzyme activity using tissues from affected organs is reportedly useful for diagnosis [103, 104]. Similarly, measuring MRC enzyme activity using renal biopsy tissue may be useful for diagnosis of mitochondrial nephropathy [105]. A 3-mm-long rapidly frozen renal biopsy specimen obtained using a 14G needle should be enough to determine MRC enzyme activity [105]. When renal biopsies are performed using 16G or 18G needles, 6 mm and 11 mm-long sections are estimated to be needed, respectively, based on the inner diameter of the biopsy needle. In nine cases of suspected mitochondrial nephropathy, we successfully measured MRC enzyme activity using 16G- or 18G-needle biopsy samples in all cases except one. Five of these patients were diagnosed with mitochondrial nephropathy, and MRC enzyme activity was reduced in four of these five patients (unpublished data). However, renal biopsy is an invasive test with a risk of hemorrhage that requires careful risk–benefit consideration.

The final diagnosis of mitochondrial nephropathy is confirmed by genetic testing. Lymphocytes from peripheral blood are typically used for these genetic tests, however, selection of cells for genetic analysis is important, particularly when pathogenic variants on mtDNA are suspected. The heteroplasmy rate of pathogenic mtDNA variants varies between different tissues/organs [2, 80, 106–109]; therefore, even if no pathogenic variants are detected on mtDNA extracted from blood cells, they may be observed on mtDNA extracted from renal tissue or urinary sediment cells, leading to diagnosis. Indeed, the heteroplasmy rate of pathogenic mtDNA variants tends to be higher in urinary sediment cells than blood cells in mitochondrial nephropathy [79]. It should also be noted that the heteroplasmy rate of pathogenic mtDNA variants in blood cells tends to decrease with age [110, 111]. In addition, genetic analysis using kidney tissue should be considered for diagnosis or investigation of disease etiology [12, 79].

The challenge is that many cases are not diagnosed even after genetic testing [102]. In some cases, the causative gene variant is not detected, even when pathology shows abnormal mitochondria, elevated lactate levels, or decreased MRC enzyme activity [6]. In such cases, the patient might not have mitochondrial disease; however, it is also possible that the pathogenic variant was undetected owing to the use of inappropriate cells for genetic analysis in cases caused by the mtDNA variant. There is also a possibility that the genetic analysis method used was inappropriate; for example, only mtDNA was analyzed in cases caused by pathological variants in nDNA, or only exome analysis was performed in cases with pathological variants in introns of genes. Moreover, it is presumed that many causative genes remain unknown.

## Differences between nephropathy secondary to mitochondrial disease and mitochondrial nephropathy

**Fig. 6:**
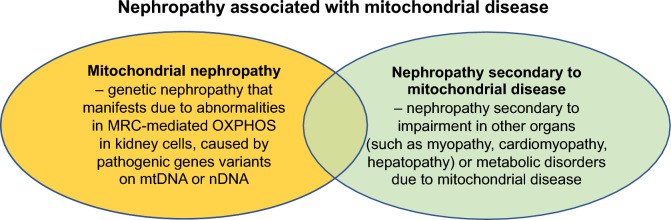
Difference between mitochondrial nephropathy and nephropathy secondary to mitochondrial disease. OXPHOS, oxidative phosphorylation; MRC, mitochondrial respiratory chain; mtDNA, mitochondrial DNA; nDNA, nuclear DNA

Mitochondrial disease can cause nephropathy even when OXPHOS in kidney cells is not genetically impaired. For example, diabetic nephropathy may develop in patients with diabetes due to mitochondrial disease [6, 79]. In some cases, myoglobin-induced tubulointerstitial damage may be caused by rhabdomyolysis due to mitochondrial myopathy and, in other cases, decreased renal function may be concomitant with decreased renal circulatory plasma flow due to cardiomyopathy [12–14]. Renal injury may also develop owing to the side-effects of drugs administered for various systemic symptoms associated with mitochondrial disease [84]. In these cases, mitochondrial function in kidney cells is not genetically impaired, and must be distinguished from mitochondrial nephropathy (Fig. 6). Nephropathy secondary to non-renal organ damage caused by mitochondrial disease requires treatment of the impaired organ or discontinuation of prescription drugs, whereas treatments in primary mitochondrial nephropathy focus on restoring mitochondrial OXPHOS function. However, in actual clinical settings, it may be difficult to distinguish between the two types of nephropathies associated with mitochondrial diseases. In such cases, the presence of renal cell pathological findings characteristic to mitochondrial nephropathy and the measurement of MRC enzyme activity using renal biopsied tissues may be useful to differentiate between primary mitochondrial nephropathies and secondary nephropathy due to mitochondrial diseases [12, 79]. In cases caused by pathogenic variants on mtDNA, heteroplasmy rates in renal tissues may be a useful criterion. For instance, if pathogenic mtDNA variants are not detected in renal tissues in a case of diabetic nephropathy with a mitochondrial disease caused by mtDNA pathogenic variants, it can be concluded that the nephropathy should be secondary due to diabetes caused by mitochondrial disease [12, 79].

## Prognosis of mitochondrial nephropathy

Few studies have assessed the prognosis of mitochondrial nephropathy based on long-term observational data. Our analysis of 63 patients with mitochondrial nephropathy due to the m.3243A>G variant showed a median rate of decline in eGFR of 5.4 (IQR: 5.0–17.0) mL/min/1.73 m^2^/year [6]. In addition, 50.8% of patients were started on renal replacement therapy at a median observation period of 11 (IQR: 5–17) years, and 25.4% patients died at a median observation period of 12 (IQR: 7–23) years [6]. It is strongly speculated that the heteroplasmy rate is a promising prognostic factor in cases with pathogenic mtDNA variants [108, 109]. Indeed, a higher heteroplasmy rate in mtDNA with the pathogenic variant in leukocytes was associated with a worse renal prognosis [109]. However, it is assumed that the association with renal prognosis is more closely related to the heteroplasmy rate of pathogenic mtDNA variant in urinary sediment cells or renal tissues.

In an analysis of 234 cases of mitochondrial nephropathy caused by pathogenic variants of genes on nDNA involved in CoQ10 synthesis, 50% of patients with pathogenic variants in *COQ2* and *COQ6* progressed to renal failure by 5 years of age, whereas no cases with pathogenic variants in *COQ8B* developed renal failure at ≤5 years of age [72]. However, more patients with the *COQ8B* pathogenic variant rapidly progressed to renal failure after the age of 5 years; at the age of 20 years, the renal survival rate was 20–25% for the *COQ2*, *COQ6*, and *COQ8B* pathogenic variants [72]. In another analysis of 35 patients with *COQ8B* pathogenic variants, a similar trend of onset at puberty followed by rapid renal failure by the age of 20 years was observed [73].

More studies based on long-term observational data are needed to fully elucidate the prognostic factors in mitochondrial nephropathy. The prognosis of mitochondrial nephropathy is expected to improve with the development and increased adoption of early diagnosis and appropriate treatment strategies.

## Treatments for mitochondrial nephropathy

The ideal treatment for mitochondrial nephropathy is the restoration of mitochondrial function in renal cells carrying the pathogenic gene variants. Although no highly efficient treatments have been identified in double-blinded clinical trials, several possible treatments have been reported.

The m.3243A>G and m.3271T>C variants inhibit taurine modification of the leucine tRNA anticodon, resulting in inaccurate recognition of the UUG codon and reduced mitochondrial protein synthesis [112, 113]. This decrease in tRNA function can be restored by external taurine replacement therapy [114]. Based on its efficacy in suppressing stroke-like episodes in MELAS associated with m.3243A>G and m.3271T>C [115], taurine has been adopted for this indication by the Medical Insurance System in Japan. Theoretically, taurine can also treat mitochondrial nephropathy caused by m.3243A>G, m.3271T>C, m.3244G>A, m.3258T>C, and m.3291T>C [115]. We previously reported one such case caused by m.3243A>G [91]. However, the effects of taurine on mitochondrial nephropathy remain unclear.

Impaired cerebral vasodilatation participates in the pathogenesis of stroke-like episodes in patients with MELAS [116]. Administration of ʟ-arginine improved vasodilatory impairment and suppressed the stroke-like episodes in MELAS [117, 118]. Therefore, ʟ-arginine may also be effective in vascular injury-type mitochondrial nephropathy (Fig. 2).

Various studies have reported the therapeutic efficacy of CoQ10 for treating mitochondrial nephropathy caused by pathogenic variants of CoQ10-related genes on nDNA [72–74, 119, 120]. In 116 patients with CoQ10-related mitochondrial nephropathy due to *COQ2*, *COQ6*, and *COQ8B* pathogenic variants who received CoQ10 (median follow-up of 2 years), there was an 88% reduction in proteinuria at 12 months compared to the untreated background-matched cohort [72]. The complete remission of proteinuria was especially apparent in patients carrying the *COQ6* pathogenic variant. CoQ10 treatment not only improved 5-year renal survival, but also improved general health and neurological symptoms, while side effects were rare and minor [72]. In other words, mitochondrial nephropathy caused by pathogenic variants of genes related to CoQ10 synthesis on nDNA requires early and lifelong CoQ10 supplementation. Doses of 10–30 mg/kg body weight/day have been effective, though the dosage may vary between cases [72, 74].

## Renal replacement therapy for patients with mitochondrial disease leading to end-stage kidney failure

Renal replacement therapy becomes necessary when mitochondrial nephropathy progresses to end-stage kidney failure, while hemodialysis, peritoneal dialysis, and kidney transplantation are candidate treatment options.

### Hemodialysis

Theoretically, and in some case reports, hemodialysis is feasible, though there have been no comprehensive reports [121, 122].

### Peritoneal dialysis

Although lactate has traditionally been included as a buffer in peritoneal dialysate solutions, high concentrations of lactate may impair physiological functions and have been replaced by peritoneal dialysate solutions containing low concentrations of lactate [123, 124]. Lactate-containing peritoneal dialysis fluid may induce lactic acidosis in patients with mitochondrial disease; however, there is no evidence of increased lactate levels [121]. There are a few case reports of patients receiving long-term peritoneal dialysis [125, 126]. Currently, no evidence renders peritoneal dialysis unsuitable for renal failure patients with mitochondrial disease; therefore, it is considered a possible renal replacement therapy of choice.

### Kidney transplantation

The first step in kidney transplantation is to determine the eligibility of the recipient with mitochondrial disease; however, there are currently no guidelines for kidney transplantation in this context. Therefore, the Kidney Disease Improving Global Outcomes (KDIGO) guidelines (https://kdigo.org/guidelines/transplant-candidate/) should be consulted.

Five cases of kidney transplantation in patients with pathogenic *COQ6* gene variants have been reported, without any recurrence after transplantation [96]; two cases with pathogenic variants in *COQ8B* also showed no recurrence for >10 years after transplantation [127], suggesting that recurrence is rare in mitochondrial nephropathy caused by pathogenic variants in CoQ10 synthesis-related genes. In addition, CoQ10 administration should be continued after transplantation. In 11 patients with the m.3243A>G variant, long-term follow-up (0.5–13 years) showed that renal function was stable in all cases after transplantation [128]. These reports indicate that kidney transplantation is a safe and effective therapy for patients with mitochondrial disease, assuming that the preoperative evaluation of recipient and donor is performed appropriately.

In the selection of live donors, particularly relatives, the KDIGO guidelines (https://kdigo.org/guidelines/living-kidney-donor/) should be consulted. Although there is no classification for mitochondrial disease, the “not graded” recommendation for the evaluation of genetic kidney diseases states that: “Donor candidates found to have a genetic kidney disease that can cause kidney failure should not donate. In cases where it remains uncertain whether the donor candidate has a genetic kidney disease and whether the disease can cause kidney failure, donation should proceed only after informing the donor candidate of the risks of donation if the disease manifests later in life.”

The mode of mtDNA inheritance and intracellular differences in heteroplasmy rates are important considerations when selecting donors from the relatives of patients developing end-stage kidney disease due to mitochondrial nephropathy with pathogenic mtDNA variants. For instance, in a mitochondrial nephropathy case with m.3243A>G, who received kidney transplantation from mother, recurrence was reported in the recipient after transplantation, and proteinuria appeared in the donor [129]. Although the cause of recurrence in the recipient and appearance of proteinuria in the donor is unclear, this case suggested that maternal donors may require especially stringent criteria in cases with pathogenic mtDNA variants, given the maternal inheritance mode of mtDNA. Although no official criteria exist for maternal donors, we provide the following recommendations: First, the presence of mitochondrial disease in the mother cannot be ruled out, especially when performing genetic testing on blood cells [79, 80, 107]. Instead, genetic tests should use urinary sediment cells [79]. Second, genetic test using renal tissue or renal pathological findings by renal biopsy should also support the donor evaluation [79, 80, 107]. However, the need for kidney biopsy to a case without decreasing renal function or proteinuria cannot be clearly stated considering the risk of the biopsy.

## Conclusion

Recent advances in genetic analysis techniques have identified various new causative genes for mitochondrial nephropathy. A fraction of patients are not complicated by symptoms of other organs, indicating that mitochondrial nephropathy may be latent in patients with symptomless chronic kidney disease. In this context, it is important to raise awareness about the disease and establish a system that facilitates diagnosis of mitochondrial nephropathy without delay. In addition, the pathogenesis of phenotypic diversity in mitochondrial nephropathy is still unclear. Further research, including basic research on mitochondrial nephropathy, is necessary to develop effective treatments in future.

## References

[1] Gorman GS, Chinnery PF, Dimauro S, et al. Mitochondrial diseases. Nat Rev Dis Primers. 2016;2:16080. 10.1038/nrdp.2016.80. 27775730 10.1038/nrdp.2016.80

[2] Russell OM, Gorman GS, Lightowlers RN, Turnbull DM. Mitochondrial diseases: hope for the future. Cell. 2020;181:168–88. 10.1016/j.cell.2020.02.051. 32220313 10.1016/j.cell.2020.02.051

[3] Wallace DC, Singh G, Lott MT, et al. Mitochondrial DNA mutation associated with Leber’s hereditary optic neuropathy. Science. 1988;242:1427–30. 10.1126/science.3201231. 3201231 10.1126/science.3201231

[4] Holt IJ, Harding AE, Morgan-Hughes JA. Deletions of muscle mitochondrial DNA in patients with mitochondrial myopathies. Nature. 1988;331:717–9. 10.1038/331717a0. 2830540 10.1038/331717a0

[5] Niaudet P, Rötig A. Renal involvement in mitochondrial cytopathies. Pediatr Nephrol. 1996;10(3):368–73. 10.1007/BF00866789. 8792408 10.1007/BF00866789

[6] Imasawa T, Hirano D, Nozu K, et al. Clinicopathologic features of mitochondrial nephropathy. Kidney Int Rep. 2022;7:580–90. 10.1016/j.ekir.2021.12.028. 35257070 10.1016/j.ekir.2021.12.028 PMC8897298

[7] Emma F, Bertini E, Salviati L, Montini G. Renal involvement in mitochondrial cytopathies. Pediatr Nephrol. 2012;27:539–50. 10.1007/s00467-011-1926-6. 21656172 10.1007/s00467-011-1926-6 PMC3288375

[8] Finsterer J, Scorza FA. Renal manifestations of primary mitochondrial disorders. Biomed Rep. 2017;6:487–94. 10.3892/br.2017.892. 28515908 10.3892/br.2017.892 PMC5431253

[9] Parasyri M, Brandström P, Uusimaa J, et al. Renal phenotype in mitochondrial diseases: a multicenter study. Kidney Dis. 2022;8:148–59. 10.1159/000521148. 10.1159/000521148 PMC9021658 35527992

[10] Yang J, Chen S, Duan F, et al. Mitochondrial cardiomyopathy: molecular epidemiology, diagnosis, models, and therapeutic management. Cells. 2022;11:3511. 10.3390/cells11213511. 36359908 10.3390/cells11213511 PMC9655095

[11] Squires JE, Miethke AG, Valencia CA, et al. Clinical spectrum and genetic causes of mitochondrial hepatopathy phenotype in children. Hepatol Commun. 2023;7: e0139. 10.1097/HC9.0000000000000139. 37184518 10.1097/HC9.0000000000000139 PMC10187840

[12] Rudnicki M, Mayr JA, Zschocke J, et al. MELAS syndrome and kidney disease without Fanconi syndrome or proteinuria: a case report. Am J Kidney Dis. 2016;68:949–53. 10.1053/j.ajkd.2016.06.027. 27683045 10.1053/j.ajkd.2016.06.027

[13] Yokoyama J, Yamaguchi H, Shigeto H, et al. A case of rhabdomyolysis after status epilepticus without stroke-like episodes in mitochondrial myopathy, encephalopathy, lactic acidosis, and stroke-like episodes. Rinsho Shinkeigaku. 2016;56:204–7. 10.5692/clinicalneurol.cn-000834. 26960270 10.5692/clinicalneurol.cn-000834

[14] Finsterer J. The spectrum of renal abnormalities in mitochondrial disorders is broad. Kidney Int Rep. 2022;7:1722. 10.1016/j.ekir.2022.05.014. 35812280 10.1016/j.ekir.2022.05.014 PMC9263252

[15] Taniike M, Fukushima H, Yanagihara I, et al. Mitochondrial tRNA(Ile) mutation in fatal cardiomyopathy. Biochem Biophys Res Commun. 1992;186:47–53. 10.1016/s0006-291x(05)80773-9. 1632786 10.1016/s0006-291x(05)80773-9

[16] Mochizuki H, Joh K, Kawame H, et al. Mitochondrial encephalomyopathies preceded by de-Toni-Debré-Fanconi syndrome or focal segmental glomerulosclerosis. Clin Nephrol. 1996;46:347–52. 8953126

[17] Tzen CY, Tsai JD, Wu TY, et al. Tubulointerstitial nephritis associated with a novel mitochondrial point mutation. Kidney Int. 2001;59:846–54. 10.1046/j.1523-1755.2001.059003846.x. 11231339 10.1046/j.1523-1755.2001.059003846.x

[18] Scaglia F, Vogel H, Hawkins EP, et al. Novel homoplasmic mutation in the mitochondrial tRNATyr gene associated with atypical mitochondrial cytopathy presenting with focal segmental glomerulosclerosis. Am J Med Genet A. 2003;123A:172–8. 10.1002/ajmg.a.20315. 14598342 10.1002/ajmg.a.20315

[19] Meulemans A, Seneca S, Lagae L, et al. A novel mitochondrial transfer RNA(Asn) mutation causing multiorgan failure. Arch Neurol. 2006;63:1194–8. 10.1001/archneur.63.8.1194. 16908752 10.1001/archneur.63.8.1194

[20] Viering D, Schlingmann KP, Hureaux M, et al. Gitelman-like syndrome caused by pathogenic variants in mtDNA. J Am Soc Nephrol. 2022;33:305–25. 10.1681/ASN.2021050596. 34607911 10.1681/ASN.2021050596 PMC8819995

[21] Goto Y, Itami N, Kajii N, et al. Renal tubular involvement mimicking Bartter syndrome in a patient with Kearns-Sayre syndrome. J Pediatr. 1990;116:904–10. 10.1016/s0022-3476(05)80648-1. 2161456 10.1016/s0022-3476(05)80648-1

[22] Eviatar L, Shanske S, Gauthier B, et al. Kearns-Sayre syndrome presenting as renal tubular acidosis. Neurology. 1990;40:1761–3. 10.1212/wnl.40.11.1761. 2234434 10.1212/wnl.40.11.1761

[23] Güçer S, Talim B, Aşan E, et al. Focal segmental glomerulosclerosis associated with mitochondrial cytopathy: report of two cases with special emphasis on podocytes. Pediatr Dev Pathol. 2005;8:710–7. 10.1007/s10024-005-0058-z. 16328667 10.1007/s10024-005-0058-z

[24] López LC, Schuelke M, Quinzii CM, et al. Leigh syndrome with nephropathy and CoQ10 deficiency due to decaprenyl diphosphate synthase subunit 2 (*PDSS2*) mutations. Am J Hum Genet. 2006;79:1125–9. 10.1086/510023. 17186472 10.1086/510023 PMC1698707

[25] Diomedi-Camassei F, Di Giandomenico S, Santorelli FM, et al. COQ2 nephropathy: a newly described inherited mitochondriopathy with primary renal involvement. J Am Soc Nephrol. 2007;18:2773–80. 10.1681/ASN.2006080833. 17855635 10.1681/ASN.2006080833

[26] Heeringa SF, Chernin G, Chaki M, et al. *COQ6* mutations in human patients produce nephrotic syndrome with sensorineural deafness. J Clin Invest. 2011;121:2013–24. 10.1172/JCI45693. 21540551 10.1172/JCI45693 PMC3083770

[27] Ashraf S, Gee HY, Woerner S, et al. *ADCK4* mutations promote steroid-resistant nephrotic syndrome through CoQ10 biosynthesis disruption. J Clin Invest. 2013;123:5179–89. 10.1172/JCI69000. 24270420 10.1172/JCI69000 PMC3859425

[28] Schijvens AM, van de Kar NC, Bootsma-Robroeks CM, et al. Mitochondrial disease and the kidney with a special focus on CoQ10 deficiency. Kidney Int Rep. 2020;5:2146–59. 10.1016/j.ekir.2020.09.044. 33305107 10.1016/j.ekir.2020.09.044 PMC7710892

[29] Lemoine S, Panaye M, Rabeyrin M, et al. Renal involvement in neuropathy, ataxia, retinitis pigmentosa (NARP) syndrome: a case report. Am J Kidney Dis. 2018;71:754–7. 10.1053/j.ajkd.2017.09.020. 29224958 10.1053/j.ajkd.2017.09.020

[30] Fervenza FC, Gavrilova RH, Nasr SH, Irazabal MV, Nath KA. CKD due to a novel mitochondrial DNA mutation: a case report. Am J Kidney Dis. 2019;73:273–7. 10.1053/j.ajkd.2018.06.032. 30309714 10.1053/j.ajkd.2018.06.032

[31] Bakis H, Trimouille A, Vermorel A, et al. Adult onset tubulo-interstitial nephropathy in MT-ND5-related phenotypes. Clin Genet. 2020;97:628–33. 10.1111/cge.13670. 31713837 10.1111/cge.13670

[32] Shayota BJ, Le NT, Bekheirnia N, et al. Characterization of the renal phenotype in RMND1-related mitochondrial disease. Mol Genet Genomic Med. 2019;7: e973. 10.1002/mgg3.973. 31568715 10.1002/mgg3.973 PMC6900359

[33] Bourdon A, Minai L, Serre V, et al. Mutation of *RRM2B*, encoding p53-controlled ribonucleotide reductase (p53R2), causes severe mitochondrial DNA depletion. Nat Genet. 2007;39:776–80. 10.1038/ng2040. 17486094 10.1038/ng2040

[34] Kanako KI, Sakakibara N, Murayama K, et al. *BCS1L* mutations produce Fanconi syndrome with developmental disability. J Hum Genet. 2022;67:143–8. 10.1038/s10038-021-00984-0. 34650211 10.1038/s10038-021-00984-0

[35] Frazier AE, Thorburn DR, Compton AG. Mitochondrial energy generation disorders: genes, mechanisms, and clues to pathology. J Biol Chem. 2019;294:5386–95. 10.1074/jbc.R117.809194. 29233888 10.1074/jbc.R117.809194 PMC6462508

[36] Murayama K, Shimura M, Liu Z, Okazaki Y, Ohtake A. Recent topics: the diagnosis, molecular genesis, and treatment of mitochondrial diseases. J Hum Genet. 2019;64:113–25. 10.1038/s10038-018-0528-6. 30459337 10.1038/s10038-018-0528-6

[37] Rahman J, Rahman S. Mitochondrial medicine in the omics era. Lancet. 2018;391:2560–74. 10.1016/S0140-6736(18)30727-X. 29903433 10.1016/S0140-6736(18)30727-X

[38] Hoefs SJG, Dieteren CEJ, Rodenburg RJ, et al. Baculovirus complementation restores a novel *NDUFAF2* mutation causing complex I deficiency. Hum Mutat. 2009;30:E728–36. 10.1002/humu.21037. 19384974 10.1002/humu.21037

[39] Leslie N, Wang X, Peng Y, et al. Neonatal multiorgan failure due to *ACAD9* mutation and complex I deficiency with mitochondrial hyperplasia in liver, cardiac myocytes, skeletal muscle, and renal tubules. Hum Pathol. 2016;49:27–32. 10.1016/j.humpath.2015.09.039. 26826406 10.1016/j.humpath.2015.09.039

[40] de Lonlay P, Valnot I, Barrientos A, et al. A mutant mitochondrial respiratory chain assembly protein causes complex III deficiency in patients with tubulopathy, encephalopathy and liver failure. Nat Genet. 2001;29:57–60. 10.1038/ng706. 11528392 10.1038/ng706

[41] Ezgu F, Senaca S, Gunduz M, et al. Severe renal tubulopathy in a newborn due to *BCS1L* gene mutation: effects of different treatment modalities on the clinical course. Gene. 2013;528:364–6. 10.1016/j.gene.2013.07.007. 23892085 10.1016/j.gene.2013.07.007

[42] Kasapkara ÇS, Tümer L, Ezgü FS, Küçükçongar A, Hasanoğlu A. *BCS1L* gene mutation causing gracile syndrome: case report. Ren Fail. 2014;36:953–4. 10.3109/0886022X.2014.900422. 24655110 10.3109/0886022X.2014.900422

[43] Tucker EJ, Wanschers BFJ, Szklarczyk R, et al. Mutations in the UQCC1-interacting protein, UQCC2, cause human complex III deficiency associated with perturbed cytochrome *b* protein expression. PLoS Genet. 2013;9: e1004034. 10.1371/journal.pgen.1004034. 24385928 10.1371/journal.pgen.1004034 PMC3873243

[44] Tay SKH, Sacconi S, Akman HO, et al. Unusual clinical presentations in four cases of Leigh disease, cytochrome *C* oxidase deficiency, and SURF1 gene mutations. J Child Neurol. 2005;20:670–4. 10.1177/08830738050200080701. 16225813 10.1177/08830738050200080701

[45] Valnot I, von Kleist-Retzow JC, Barrientos A, et al. A mutation in the human heme A:farnesyltransferase gene (*COX10*) causes cytochrome *c* oxidase deficiency. Hum Mol Genet. 2000;9:1245–9. 10.1093/hmg/9.8.1245. 10767350 10.1093/hmg/9.8.1245

[46] Honzík T, Tesarová M, Mayr JA, et al. Mitochondrial encephalocardio-myopathy with early neonatal onset due to *TMEM70* mutation. Arch Dis Child. 2010;95:296–301. 10.1136/adc.2009.168096. 20335238 10.1136/adc.2009.168096

[47] Magner M, Dvorakova V, Tesarova M, et al. Erratum to: TMEM70 deficiency: long-term outcome of 48 patients. J Inherit Metab Dis. 2015;38:583–4. 10.1007/s10545-015-9833-9. 25778942 10.1007/s10545-015-9833-9

[48] Dimmock DP, Zhang Q, Dionisi-Vici C, et al. Clinical and molecular features of mitochondrial DNA depletion due to mutations in deoxyguanosine kinase. Hum Mutat. 2008;29:330–1. 10.1002/humu.9519. 18205204 10.1002/humu.9519

[49] Morava E, Steuerwald U, Carrozzo R, et al. Dystonia and deafness due to *SUCLA2* defect; Clinical course and biochemical markers in 16 children. Mitochondrion. 2009;9:438–42. 10.1016/j.mito.2009.08.003. 19666145 10.1016/j.mito.2009.08.003

[50] Carrozzo R, Bornstein B, Lucioli S, et al. Mutation analysis in 16 patients with mtDNA depletion. Hum Mutat. 2003;21:453–4. 10.1002/humu.9135. 12655576 10.1002/humu.9135

[51] El-Hattab AW, Scaglia F. Mitochondrial DNA depletion syndromes: review and updates of genetic basis, manifestations, and therapeutic options. Neurotherapeutics. 2013;10:186–98. 10.1007/s13311-013-0177-6. 23385875 10.1007/s13311-013-0177-6 PMC3625391

[52] Shoffner JM, Voljavec AS, Dixon J, et al. Renal amino acid transport in adults with oxidative phosphorylation diseases. Kidney Int. 1995;47:1101–7. 10.1038/ki.1995.157. 7783407 10.1038/ki.1995.157

[53] Belostotsky R, Ben-Shalom E, Rinat C, et al. Mutations in the mitochondrial seryl-tRNA synthetase cause hyperuricemia, pulmonary hypertension, renal failure in infancy and alkalosis, HUPRA syndrome. Am J Hum Genet. 2011;88:193–200. 10.1016/j.ajhg.2010.12.010. 21255763 10.1016/j.ajhg.2010.12.010 PMC3035710

[54] Rivera H, Martín-Hernández E, Delmiro A, et al. A new mutation in the gene encoding mitochondrial seryl-tRNA synthetase as a cause of HUPRA syndrome. BMC Nephrol. 2013;14:195. 10.1186/1471-2369-14-195. 24034276 10.1186/1471-2369-14-195 PMC3847196

[55] Nakajima J, Eminoglu TF, Vatansever G, et al. A novel homozygous *YARS2* mutation causes severe myopathy, lactic acidosis, and sideroblastic anemia 2. J Hum Genet. 2014;59:229–32. 10.1038/jhg.2013.143. 24430573 10.1038/jhg.2013.143

[56] Riley LG, Rudinger-Thirion J, Schmitz-Abe K, et al. LARS2 variants associated with hydrops, lactic Acidosis, sideroblastic anemia, and multisystem failure. JIMD Rep. 2016;28:49–57. 10.1007/8904_2015_515. 26537577 10.1007/8904_2015_515 PMC5059179

[57] Saada A, Shaag A, Arnon S, et al. Antenatal mitochondrial disease caused by mitochondrial ribosomal protein (*MRPS22*) mutation. J Med Genet. 2007;44:784–6. 10.1136/jmg.2007.053116. 17873122 10.1136/jmg.2007.053116 PMC2652816

[58] Distelmaier F, Haack TB, Catarino CB, et al. *MRPL44* mutations cause a slowly progressive multisystem disease with childhood-onset hypertrophic cardiomyopathy. Neurogenetics. 2015;16:319–23. 10.1007/s10048-015-0444-2. 25797485 10.1007/s10048-015-0444-2

[59] Vedrenne V, Galmiche L, Chretien D, et al. Mutation in the mitochondrial translation elongation factor EFTs results in severe infantile liver failure. J Hepatol. 2012;56:294–7. 10.1016/j.jhep.2011.06.014. 21741925 10.1016/j.jhep.2011.06.014

[60] O’Toole JF, Liu Y, Davis EE, et al. Individuals with mutations in *XPNPEP3*, which encodes a mitochondrial protein, develop a nephronophthisis-like nephropathy. J Clin Invest. 2010;120:791–802. 10.1172/JCI40076. 20179356 10.1172/JCI40076 PMC2827951

[61] Kanabus M, Shahni R, Saldanha JW, et al. Bi-allelic *CLPB* mutations cause cataract, renal cysts, nephrocalcinosis and 3-methylglutaconic aciduria, a novel disorder of mitochondrial protein disaggregation. J Inherit Metab Dis. 2015;38:211–9. 10.1007/s10545-015-9813-0. 25595726 10.1007/s10545-015-9813-0

[62] Dweikat I, Naser E, Damsah N, Libdeh BA, Bakri I. Ethylmalonic encephalopathy associated with crescentic glomerulonephritis. Metab Brain Dis. 2012;27:613–6. 10.1007/s11011-012-9313-y. 22584649 10.1007/s11011-012-9313-y

[63] Duncan AJ, Bitner-Glindzicz M, Meunier B, et al. A nonsense mutation in *COQ9* causes autosomal-recessive neonatal-onset primary coenzyme Q10 deficiency: a potentially treatable form of mitochondrial disease. Am J Hum Genet. 2009;84:558–66. 10.1016/j.ajhg.2009.03.018. 19375058 10.1016/j.ajhg.2009.03.018 PMC2681001

[64] Rahman S, Hargreaves I, Clayton P, Heales S. Neonatal presentation of coenzyme Q10 deficiency. J Pediatr. 2001;139:456–8. 10.1067/mpd.2001.117575. 11562630 10.1067/mpd.2001.117575

[65] Manwaring N, Jones MM, Wang JJ, et al. Population prevalence of the MELAS *A3243G* mutation. Mitochondrion. 2007;7:230–3. 10.1016/j.mito.2006.12.004. 17300999 10.1016/j.mito.2006.12.004

[66] Duann P, Lin PH. Mitochondria damage and kidney disease. Adv Exp Med Biol. 2017;982:529–51. 10.1007/978-3-319-55330-6_27. 28551805 10.1007/978-3-319-55330-6_27 PMC8049117

[67] Imasawa T, Rossignol R. Podocyte energy metabolism and glomerular diseases. Int J Biochem Cell Biol. 2013;45:2109–18. 10.1016/j.biocel.2013.06.013. 23806869 10.1016/j.biocel.2013.06.013

[68] Hagiwara M, Yamagata K, Capaldi RA, Koyama A. Mitochondrial dysfunction in focal segmental glomerulosclerosis of puromycin aminonucleoside nephrosis. Kidney Int. 2006;69:1146–52. 10.1038/sj.ki.5000207. 16609681 10.1038/sj.ki.5000207

[69] Kaneko S, Usui J, Hagiwara M, et al. Mitochondrial DNA deletion-dependent podocyte injuries in Mito-miceΔ, a murine model of mitochondrial disease. Exp Anim. 2022;71:14–21. 10.1538/expanim.21-0054. 34321368 10.1538/expanim.21-0054 PMC8828406

[70] Govers LP, Toka HR, Hariri A, Walsh SB, Bockenhauer D. Mitochondrial DNA mutations in renal disease: an overview. Pediatr Nephrol. 2021;36:9–17. 10.1007/s00467-019-04404-6. 31925537 10.1007/s00467-019-04404-6 PMC7701126

[71] Wilson FH, Hariri A, Farhi A, et al. A cluster of metabolic defects caused by mutation in a mitochondrial tRNA. Science. 2004;306:1190–4. 10.1126/science.1102521. 15498972 10.1126/science.1102521 PMC3033655

[72] Drovandi S, Lipska-Ziętkiewicz BS, Ozaltin F, et al. Variation of the clinical spectrum and genotype-phenotype associations in coenzyme Q10 deficiency associated glomerulopathy. Kidney Int. 2022;102:592–603. 10.1016/j.kint.2022.02.040. 35483523 10.1016/j.kint.2022.02.040

[73] Korkmaz E, Lipska-Ziętkiewicz BS, Boyer O, et al. ADCK4-associated glomerulopathy causes adolescence-onset FSGS. J Am Soc Nephrol. 2016;27:63–8. 10.1681/ASN.2014121240. 25967120 10.1681/ASN.2014121240 PMC4696579

[74] Maeoka Y, Doi T, Aizawa M, et al. A case report of adult-onset COQ8B nephropathy presenting focal segmental glomerulosclerosis with granular swollen podocytes. BMC Nephrol. 2020;21:376. 10.1186/s12882-020-02040-z. 32859164 10.1186/s12882-020-02040-z PMC7456044

[75] Massin P, Dubois-Laforgue D, Meas T, et al. Retinal and renal complications in patients with a mutation of mitochondrial DNA at position 3243 (maternally inherited diabetes and deafness). A case-control study. Diabetologia. 2008;51:1664–70. 10.1007/s00125-008-1073-1. 18581092 10.1007/s00125-008-1073-1

[76] Hall AM, Vilasi A, Garcia-Perez I, et al. The urinary proteome and metabonome differ from normal in adults with mitochondrial disease. Kidney Int. 2015;87:610–22. 10.1038/ki.2014.297. 25207879 10.1038/ki.2014.297

[77] Zhao T, Goedhart C, Pfeffer G, et al. Skeletal phenotypes due to abnormalities in mitochondrial protein homeostasis and import. Int J Mol Sci. 2020;21:8327. 10.3390/ijms21218327. 33171986 10.3390/ijms21218327 PMC7664180

[78] Bargagli M, Primiano G, Primiano A, et al. Recurrent kidney stones in a family with a mitochondrial disorder due to the m.3243A>G mutation. Urolithiasis. 2019;47:489–92. 10.1007/s00240-018-1087-1. 30406307 10.1007/s00240-018-1087-1

[79] Imasawa T, Murayama K. In reply to “The spectrum of renal abnormalities in mitochondrial disorders is broad.” Kidney Int Rep. 2022;7:1723–4. 10.1016/j.ekir.2022.05.015. 35812298 10.1016/j.ekir.2022.05.015 PMC9263201

[80] Moreira JD, Gopal DM, Kotton DN, Fetterman JL. Gaining insight into mitochondrial genetic variation and downstream pathophysiology: What can i(PSCs) do? Genes. 2021;12:1668. 10.3390/genes12111668. 34828274 10.3390/genes12111668 PMC8624338

[81] Kobayashi A, Goto Y, Nagata M, Yamaguchi Y. Granular swollen epithelial cells: a histologic and diagnostic marker for mitochondrial nephropathy. Am J Surg Pathol. 2010;34:262–70. 10.1097/PAS.0b013e3181cb4ed3. 20090504 10.1097/PAS.0b013e3181cb4ed3

[82] Yamagata K, Muro K, Usui J, et al. Mitochondrial DNA mutations in focal segmental glomerulosclerosis lesions. J Am Soc Nephrol. 2002;13:1816–23. 10.1097/01.asn.0000019772.17954.f8. 12089377 10.1097/01.asn.0000019772.17954.f8

[83] Bakis H, Trimouille A, Vermorel A, et al. Renal involvement is frequent in adults with primary mitochondrial disorders: an observational study. Clin Kidney J. 2023;16:100–10. 10.1093/ckj/sfac195. 36726431 10.1093/ckj/sfac195 PMC9871853

[84] Viering DHHM, Vermeltfoort L, Bindels RJM, Deinum J, de Baaij JHF. Electrolyte disorders in mitochondrial cytopathies: a systematic review. J Am Soc Nephrol. 2023;34:1875–88. 10.1681/ASN.0000000000000224. 37678265 10.1681/ASN.0000000000000224 PMC10631606

[85] Hotta O, Inoue CN, Miyabayashi S, et al. Clinical and pathologic features of focal segmental glomerulosclerosis with mitochondrial tRNA^Leu(UUR)^ gene mutation. Kidney Int. 2001;59:1236–43. 10.1046/j.1523-1755.2001.0590041236.x. 11260383 10.1046/j.1523-1755.2001.0590041236.x

[86] Arbustini E, Diegoli M, Fasani R, et al. Mitochondrial DNA mutations and mitochondrial abnormalities in dilated cardiomyopathy. Am J Pathol. 1998;153:1501–10. 10.1016/S0002-9440(10)65738-0. 9811342 10.1016/S0002-9440(10)65738-0 PMC1853408

[87] Hazard FK, Ficicioglu CH, Ganesh J, Ruchelli ED. Liver pathology in infantile mitochondrial DNA depletion syndrome. Pediatr Dev Pathol. 2013;16:415–24. 10.2350/12-07-1229-OA.1. 24050659 10.2350/12-07-1229-OA.1

[88] Vincent AE, Ng YS, White K, et al. The spectrum of mitochondrial ultrastructural defects in mitochondrial myopathy. Sci Rep. 2016;6:30610. 10.1038/srep30610. 27506553 10.1038/srep30610 PMC4978969

[89] Mori K, Narahara K, Ninomiya S, Goto Y, Nonaka I. Renal and skin involvement in a patient with complete Kearns-Sayre syndrome. Am J Med Genet. 1991;38:583–7. 1648309 10.1002/ajmg.1320380417

[90] Herlitz LC, Mohan S, Stokes MB, et al. Tenofovir nephrotoxicity: acute tubular necrosis with distinctive clinical, pathological, and mitochondrial abnormalities. Kidney Int. 2010;78:1171–7. 10.1038/ki.2010.318. 20811330 10.1038/ki.2010.318

[91] Imasawa T, Kitamura H, Kawaguchi T, et al. Changes in histopathology and heteroplasmy rates over 8 years and effectiveness of taurine supplementation in a patient with mitochondrial nephropathy caused by *MT-TL1* mutation: a case report. Heliyon. 2023;9: e14923. 10.1016/j.heliyon.2023.e14923. 37082626 10.1016/j.heliyon.2023.e14923 PMC10112021

[92] Naganuma T, Imasawa T, Nukui I, et al. Focal segmental glomerulosclerosis with a mutation in the mitochondrially encoded NADH dehydrogenase 5 gene: a case report. Mol Genet Metab Rep. 2023;35: 100963. 10.1016/j.ymgmr.2023.100963. 36941957 10.1016/j.ymgmr.2023.100963 PMC10024046

[93] Doleris LM, Hill GS, Chedin P, et al. Focal segmental glomerulosclerosis associated with mitochondrial cytopathy. Kidney Int. 2000;58:1851–8. 10.1111/j.1523-1755.2000.00356.x. 11044204 10.1111/j.1523-1755.2000.00356.x

[94] Connor TM, Hoer S, Mallett A, et al. Mutations in mitochondrial DNA causing tubulointerstitial kidney disease. PLoS Genet. 2017;13: e1006620. 10.1371/journal.pgen.1006620. 28267784 10.1371/journal.pgen.1006620 PMC5360345

[95] Emma F, Salviati L. Mitochondrial cytopathies and the kidney. Nephrol Ther. 2017;13(Suppl 1):S23–8. 10.1016/j.nephro.2017.01.014. 28577739 10.1016/j.nephro.2017.01.014

[96] Park E, Ahn YH, Kang HG, et al. *COQ6* mutations in children with steroid-resistant focal segmental glomerulosclerosis and sensorineural hearing loss. Am J Kidney Dis. 2017;70:139–44. 10.1053/j.ajkd.2016.10.040. 28117207 10.1053/j.ajkd.2016.10.040

[97] Hara S, Ishimura T, Fujisawa M, Nishi S, Itoh T. Granular swollen epithelial cells in the kidney allograft: a clinicopathological study with special emphasis on possible marker for kidney allograft aging. Nephrology (Carlton). 2016;21(Suppl 1):14–9. 10.1111/nep.12764. 26969019 10.1111/nep.12764

[98] Imasawa T, Tanaka M, Maruyama N, et al. Pathological similarities between low birth weight-related nephropathy and nephropathy associated with mitochondrial cytopathy. Diagn Pathol. 2014;9:181. 10.1186/s13000-014-0181-0. 25350944 10.1186/s13000-014-0181-0 PMC4189739

[99] Robinson BH. Lactic acidemia and mitochondrial disease. Mol Genet Metab. 2006;89:3–13. 10.1016/j.ymgme.2006.05.015. 16854608 10.1016/j.ymgme.2006.05.015

[100] Fujita Y, Ito M, Kojima T, et al. GDF15 is a novel biomarker to evaluate efficacy of pyruvate therapy for mitochondrial diseases. Mitochondrion. 2015;20:34–42. 10.1016/j.mito.2014.10.006. 25446397 10.1016/j.mito.2014.10.006

[101] Koga Y, Povalko N, Inoue E, et al. A new diagnostic indication device of a biomarker growth differentiation factor 15 for mitochondrial diseases: from laboratory to automated inspection. J Inherit Metab Dis. 2021;44:358–66. 10.1002/jimd.12317. 32965044 10.1002/jimd.12317 PMC8048444

[102] Ogawa E, Shimura M, Fushimi T, et al. Clinical validity of biochemical and molecular analysis in diagnosing Leigh syndrome: a study of 106 Japanese patients. J Inherit Metab Dis. 2017;40:685–93. 10.1007/s10545-017-0042-6. 28429146 10.1007/s10545-017-0042-6 PMC5579154

[103] Shimura M, Kuranobu N, Ogawa-Tominaga M, et al. Clinical and molecular basis of hepatocerebral mitochondrial DNA depletion syndrome in Japan: evaluation of outcomes after liver transplantation. Orphanet J Rare Dis. 2020;15:169. 10.1186/s13023-020-01441-5. 32703289 10.1186/s13023-020-01441-5 PMC7379809

[104] Maruo Y, Ueda Y, Murayama K, Takeda A. A case report of Leigh syndrome diagnosed by endomyocardial biopsy. Eur Heart J Case Rep. 2021;5:ytaa582. 10.1093/ehjcr/ytaa582. 33644659 10.1093/ehjcr/ytaa582 PMC7898571

[105] Ghose A, Taylor CM, Howie AJ, et al. Measurement of respiratory chain enzyme activity in human renal biopsy specimens. J Clin Med. 2017;6:90. 10.3390/jcm6090090. 28925945 10.3390/jcm6090090 PMC5615283

[106] Leonard JV, Schapira AH. Mitochondrial respiratory chain disorders I: mitochondrial DNA defects. Lancet. 2000;355:299–304. 10.1016/s0140-6736(99)05225-3. 10675086 10.1016/s0140-6736(99)05225-3

[107] Finsterer J. Mitochondriopathies. Eur J Neurol. 2004;11:163–86. 10.1046/j.1351-5101.2003.00728.x. 15009163 10.1046/j.1351-5101.2003.00728.x

[108] Grady JP, Pickett SJ, Ng YS, et al. mtDNA heteroplasmy level and copy number indicate disease burden in m.3243A>G mitochondrial disease. EMBO Mol Med. 2018;10: e8262. 10.15252/emmm.201708262. 29735722 10.15252/emmm.201708262 PMC5991564

[109] Shand JAD, Potter HC, Pilmore HL, Cundy T, Murphy R. Increased peripheral blood heteroplasmy of the mt.3243A>G mutation is associated with earlier end-stage kidney disease: a case report and review of the literature. Nephron. 2020;144:358–62. 10.1159/000507732. 32434190 10.1159/000507732

[110] Franco M, Pickett SJ, Fleischmann Z, et al. Dynamics of the most common pathogenic mtDNA variant m.3243A>G demonstrate frequency-dependency in blood and positive selection in the germline. Hum Mol Genet. 2022;31:4075–86. 10.1093/hmg/ddac149. 35849052 10.1093/hmg/ddac149 PMC9703810

[111] Rajasimha HK, Chinnery PF, Samuels DC. Selection against pathogenic mtDNA mutations in a stem cell population leads to the loss of the 3243A→G mutation in blood. Am J Hum Genet. 2008;82:333–43. 10.1016/j.ajhg.2007.10.007. 18252214 10.1016/j.ajhg.2007.10.007 PMC2427290

[112] Yasukawa T, Suzuki T, Ueda T, Ohta S, Watanabe K. Modification defect at anticodon wobble nucleotide of mitochondrial tRNAs^(Leu)(UUR)^ with pathogenic mutations of mitochondrial myopathy, encephalopathy, lactic acidosis, and stroke-like episodes. J Biol Chem. 2000;275:4251–7. 10.1074/jbc.275.6.4251. 10660592 10.1074/jbc.275.6.4251

[113] Suzuki T, Suzuki T, Wada T, Saigo K, Watanabe K. Taurine as a constituent of mitochondrial tRNAs: new insights into the functions of taurine and human mitochondrial diseases. EMBO J. 2002;21:6581–9. 10.1093/emboj/cdf656. 12456664 10.1093/emboj/cdf656 PMC136959

[114] Rikimaru M, Ohsawa Y, Wolf AM, et al. Taurine ameliorates impaired the mitochondrial function and prevents stroke-like episodes in patients with MELAS. Intern Med. 2012;51:3351–7. 10.2169/internalmedicine.51.7529. 23257519 10.2169/internalmedicine.51.7529

[115] Ohsawa Y, Hagiwara H, Nishimatsu SI, et al. Taurine supplementation for prevention of stroke-like episodes in MELAS: a multicentre, open-label, 52-week phase III trial. J Neurol Neurosurg Psychiatry. 2019;90:529–36. 10.1136/jnnp-2018-317964. 29666206 10.1136/jnnp-2018-317964 PMC6581075

[116] Nishioka J, Akita Y, Yatsuga S, et al. Inappropriate intracranial hemodynamics in the natural course of MELAS. Brain Dev. 2008;30:100–5. 10.1016/j.braindev.2007.06.008. 17664050 10.1016/j.braindev.2007.06.008

[117] Koga Y, Akita Y, Nishioka J, et al. L-arginine improves the symptoms of stroke like episodes in MELAS. Neurology. 2005;64:710–2. 10.1212/01.WNL.0000151976.60624.01. 15728297 10.1212/01.WNL.0000151976.60624.01

[118] Koga Y, Akita Y, Junko N, et al. Endothelial dysfunction in MELAS improved by l-arginine supplementation. Neurology. 2006;66:1766–9. 10.1212/01.wnl.0000220197.36849.1e. 16769961 10.1212/01.wnl.0000220197.36849.1e

[119] Atmaca M, Gulhan B, Korkmaz E, et al. Follow-up results of patients with *ADCK4* mutations and the efficacy of CoQ10 treatment. Pediatr Nephrol. 2017;32:1369–75. 10.1007/s00467-017-3634-3. 28337616 10.1007/s00467-017-3634-3

[120] Nakanishi K, Okamoto T, Nozu K, et al. Pair analysis and custom array CGH can detect a small copy number variation in *COQ6* gene. Clin Exp Nephrol. 2019;23:669–75. 10.1007/s10157-018-1682-z. 30584653 10.1007/s10157-018-1682-z

[121] Tomonari H, Yoshida H, Omura K, et al. An autopsy case of mitochondrial myopathy, encephalopathy, lactic acidosis and stroke-like episodes (MELAS) undergoing long-term hemodialysis. Nihon Toseki Igakkai Zasshi. 1996;29:1097–102. 10.4009/jsdt.29.1097.

[122] Yanagihara C, Oyama A, Tanaka M, Nakaji K, Nishimura Y. An autopsy case of mitochondrial encephalomyopathy with lactic acidosis and stroke-like episodes syndrome with chronic renal failure. Intern Med. 2001;40:662–5. 10.2169/internalmedicine.40.662. 11506313 10.2169/internalmedicine.40.662

[123] Plum J, Razeghi P, Lordnejad RM, et al. Peritoneal dialysis fluids with a physiologic pH based on either lactate or bicarbonate buffer-effects on human mesothelial cells. Am J Kidney Dis. 2001;38:867–75. 10.1053/ajkd.2001.27709. 11576893 10.1053/ajkd.2001.27709

[124] Hoshino T, Kaneko S, Minato S, et al. Longer-period effects of bicarbonate/lactate-buffered neutral peritoneal dialysis fluid in patients undergoing peritoneal dialysis. Ther Apher Dial. 2018;22:641–8. 10.1111/1744-9987.12709. 30014626 10.1111/1744-9987.12709

[125] Watanabe M, Nakata A, Takahashi S, et al. A family demonstrating two cases (mother and daughter) of mitochondrial encephalomyopathy (MELAS) with end-stage renal failure treated by long-term CAPD. Nihon Toseki Igakkai Zasshi. 2004;37:151–6. 10.4009/jsdt.37.151.

[126] Ariaudo C, Daidola G, Ferrero B, et al. Mitochondrial neurogastrointestinal encephalomyopathy treated with peritoneal dialysis and bone marrow transplantation. J Nephrol. 2015;28:125–7. 10.1007/s40620-014-0069-9. 24599829 10.1007/s40620-014-0069-9

[127] Wang S, Zhao F, Li L, Yu Z. Long-term nephrotic syndrome recurrence risk of kidney transplantation in two siblings with ADCK4-associated glomerulopathy. Pediatr Transplant. 2022;26: e14143. 10.1111/petr.14143. 34605136 10.1111/petr.14143

[128] de Laat P, van Engelen N, Wetzels JF, Smeitink JAM, Janssen MCH. Five non-mitochondrial myopathy, encephalopathy, lactic acidosis and stroke-like episodes phenotype adult patients with m.3243A>G mutation after kidney transplantation: follow-up and review of the literature. Clin Kidney J. 2019;12:840–6. 10.1093/ckj/sfz020. 31807297 10.1093/ckj/sfz020 PMC6885678

[129] Nishida H, Nawano T, Fukuhara H, et al. Outcomes of living kidney transplantation for mitochondrial disease patients: a case series. Transplant Proc. 2022;54:267–71. 10.1016/j.transproceed.2021.12.033. 35074160 10.1016/j.transproceed.2021.12.033

