# Correction: Comprehensive review of mitochondrial nephropathy—a renal phenotype in mitochondrial disease: causative genes, clinical and pathological features, diagnosis, prognosis, and treatment

**DOI:** 10.1007/s10157-025-02665-0

**Published:** 2025-03-21

**Authors:** Toshiyuki Imasawa, Kei Murayama, Daishi Hirano, Kandai Nozu

**Affiliations:** 1 Department of Nephrology, National Hospital Organization Chibahigashi National Hospital, 673 Nitona-cho, Chuoh-ku, Chiba, 206-8712 Japan; 2 Diagnostics and Therapeutic of Intractable Diseases, Intractable Disease Research Center, Graduate School of Medicine, Juntendo University, 2-1-1, Hongo, Bunkyo-ku, Tokyo, 113-8421 Japan; 3 Department of Pediatrics, The Jikei University School of Medicine, 3-25-8 Nishi-Shinbashi, Minato-ku, Tokyo, 105-0003 Japan; 4 Department of Pediatrics, Kobe University Graduate School of Medicine, Kobe, Hyogo 650-0017 Japan

**Correction: Clinical and Experimental Nephrology (2025) 29:39–56** 10.1007/s10157-024-02554-y

The article “Comprehensive review of mitochondrial nephropathy—a renal phenotype in mitochondrial disease: causative genes, clinical and pathological features, diagnosis, prognosis, and treatment”, written by Toshiyuki Imasawa, Kei Murayama, Daishi Hirano, Kandai Nozu was originally published under © The Author(s), under exclusive licence to Japanese Society of Nephrology 2024. As a result of the subsequent decision to publish the article under the open access model, the article’s copyright notice was changed on 04 March 2025 to © The Author(s) 2025 and the article is now distributed under a Creative Commons Attribution 4.0 International License, which permits use, sharing, adaptation, distribution and reproduction in any medium or format, as long as you give appropriate credit to the original author(s) and the source, provide a link to the Creative Commons licence, and indicate if changes were made. The images or other third party material in this article are included in the article’s Creative Commons licence, unless indicated otherwise in a credit line to the material. If material is not included in the article’s Creative Commons licence and your intended use is not permitted by statutory regulation or exceeds the permitted use, you will need to obtain permission directly from the copyright holder. To view a copy of this licence, visit http://creativecommons.org/licenses/by/4.0.

